# Supplementary material for: Role of iRhom2 in Olfaction: Implications for Odorant Receptor Regulation and Activity-Dependent Adaptation
Source: Int J Mol Sci. 2024 May 31;25(11):6079. doi: 10.3390/ijms25116079 (PMC11173328; doi:10.3390/ijms25116079)
Supplement: Supplementary file 1 [file ijms-25-06079-s001.zip › ijms-2978120-supplementary.pdf]

Supplementary Figures for the manuscript entitled
Role of iRhom2 in Olfaction: Implications for Odorant Receptor Regulation and Activity-dependent Adaptation, by Azzopardi, Stephanie et al.

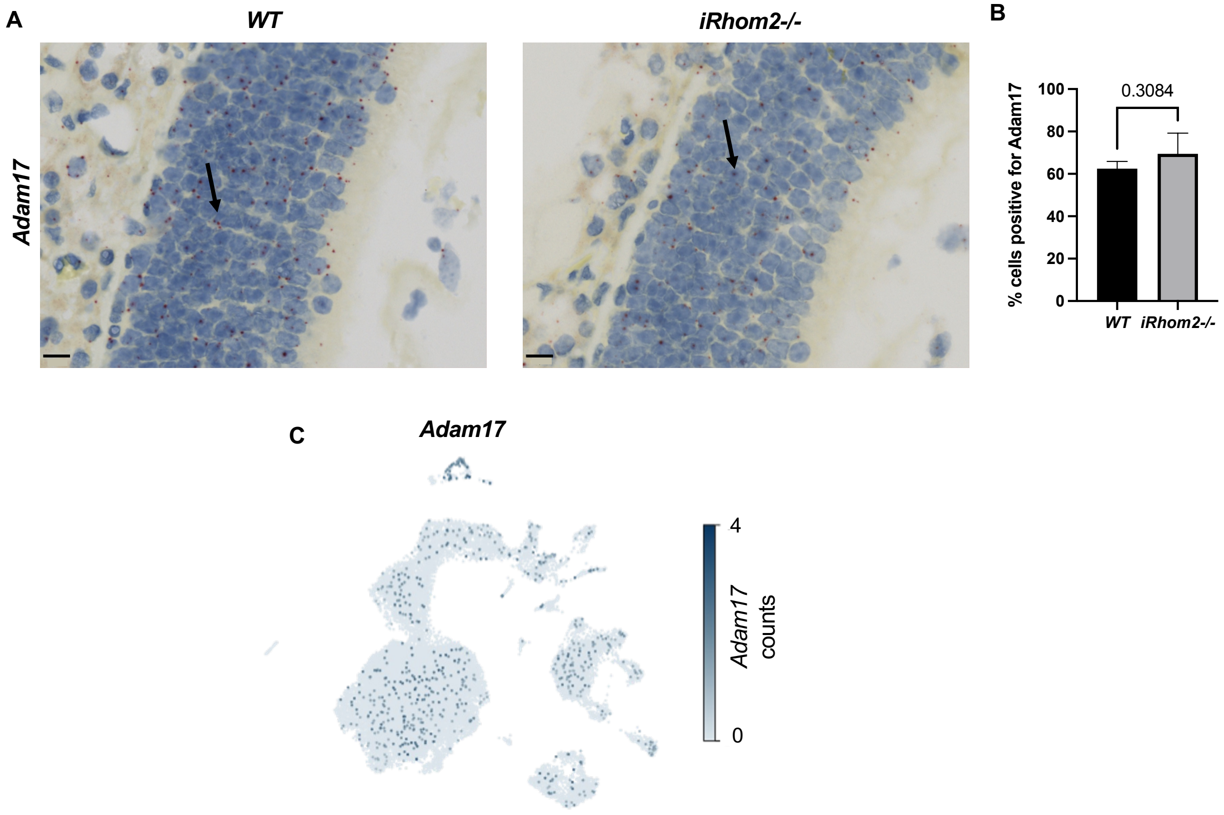

Supplementary Figure S1. Expression of ADAM17 in the OE. A, B) RNA in situ hybridization with probe Mm-Adam17 of *WT* and *iRhom2-/-* olfactory epithelium reveals the expression pattern of *Adam17* (A, scalebars = 10 µm, indicated by black arrows), which is present at similar levels in the OSNs of both genotypes (quantification shown in B). C) Single cell RNAseq analysis shows ubiquitous expression of *Adam17* in diverse OE cell types corresponding to those labeled in Figure 1F.

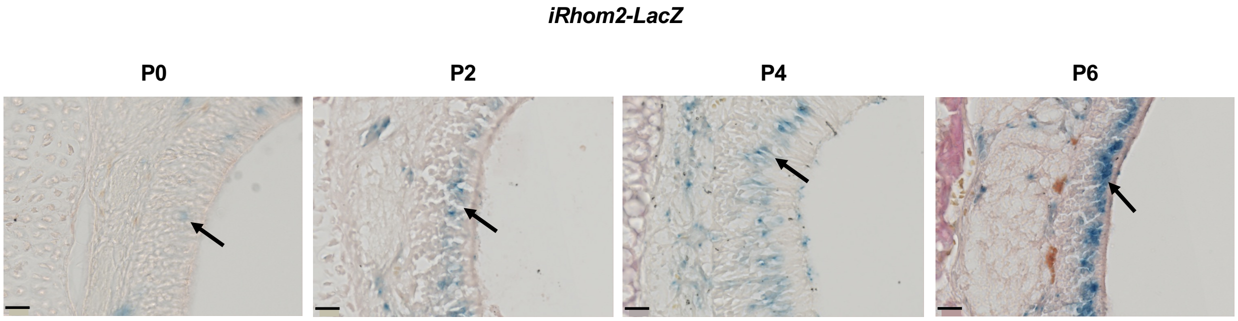

Supplementary Figure S2. Expression of *iRhom2* at birth and up to 6 days of age. An analysis of iRhom2-LacZ expression (black arrows) demonstrated little, if any expression at birth, but clearly detectable expression at postnatal day 2 (P2) and increasing expression at P4 and P6 (scalebars = 20 μm).

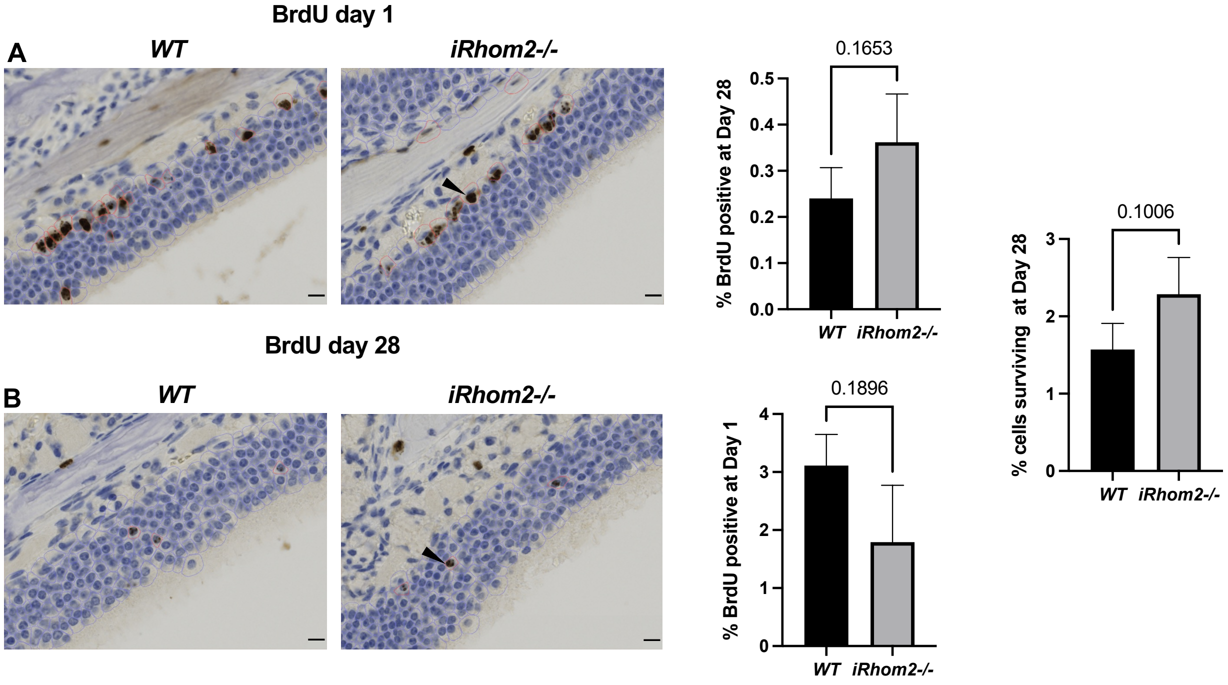

Supplementary Figure S3. Analysis of BrdU incorporation into the OE at 1 day or 28 days after BrdU injection into mice. A) Olfactory epithelium of mice injected 24 hours prior with BrdU revealed similar levels of neurogenesis between genotypes. B) After 28 days, *iRhom2-/-* OSNs trended toward longer survival, but the difference between genotypes was not significant. All scalebars = 10 μm; *n* = 3 per group, black arrowheads indicate BrDU staining.

A

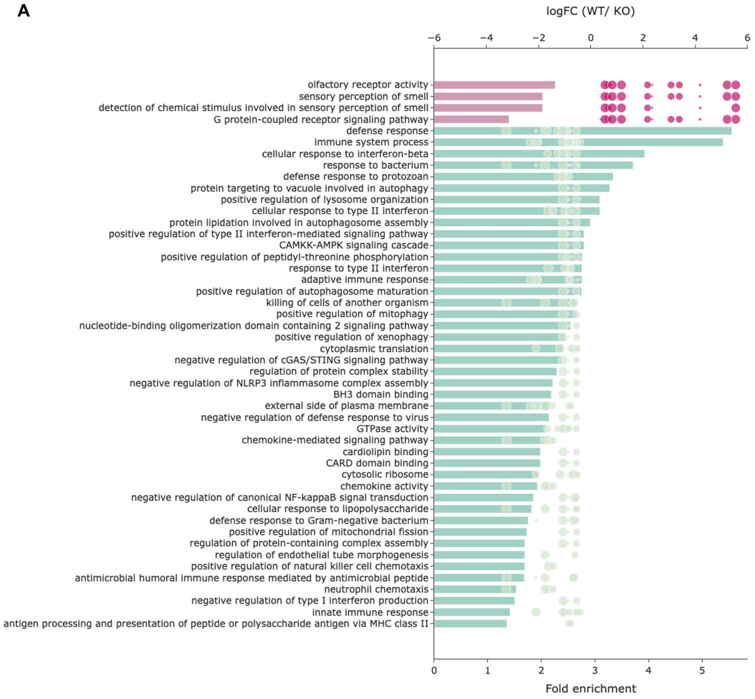

B

Up-regulated in *iRhom2 -/-*
GO terms

Olfr603
Olfr323
Olfr331
Olfr726
Olfr171
Olfr536
Olfr585
Olfr564
Olfr568
Olfr287
Olfr543
Gpr162

Down-regulated in *iRhom2-/-*
GO terms

Irgm1
Cxcl10
Ifi47
Dmbt1
Igtp
Tgtp2
Gm12185
Cxcl9
Irgm2
Gbp4
Arid5a
Ttll12
Slfn2
Cd8a
Cd8b1
H2-Aa
Cd79b
Slamf1
H2-Eb1
Apobec3
Bank1
Gbp9
Ccl5
Ccl7
Chil1
Ciita
Hmgn2
Rpl30
Rps2
Rpl3
Rps15a
Rps13
Klrc1
Il2rb
Hdac1
Fgf1

Supplementary Figure S4. Complete bulk RNAseq data GO analysis. A) The complete list of GO terms, including many related to downregulation of innate immune function in the *iRhom2-/-* OE. B) The genes contributing to the upregulated GO terms reveals 11 ORs and 1 non-OR, while the genes contributing to the downregulated GO terms are non-ORs, many of which are related to immunity. (*n* = 12 per genotype)

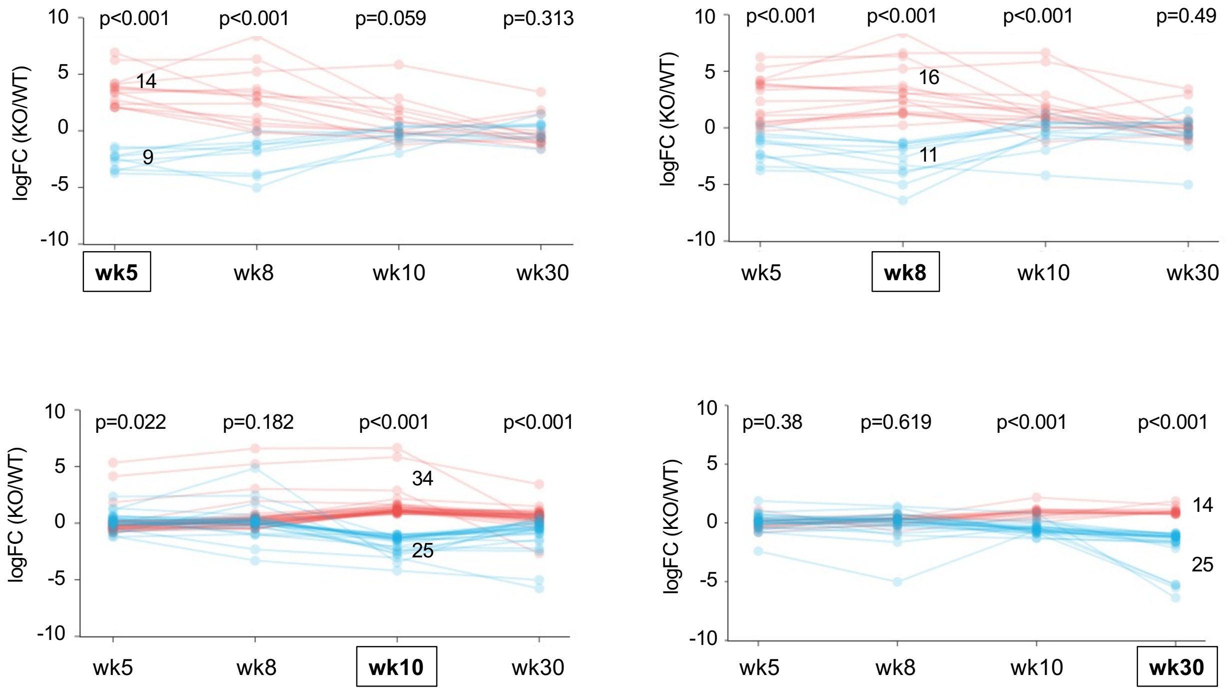

Supplementary Figure S5. DE ORs plotted by age. The number of upregulated (red) and downregulated (blue) ORs at each age (n = 3 per genotype, age indicated by box) as determined by bulk RNASeq is indicated by the numbers over the line graphs. The DE ORs from the indicated age (DE-FDR <0.05, n = 3) are graphed over time to visualize changes in logFC. Results of Student's t-tests are indicated above each time point.

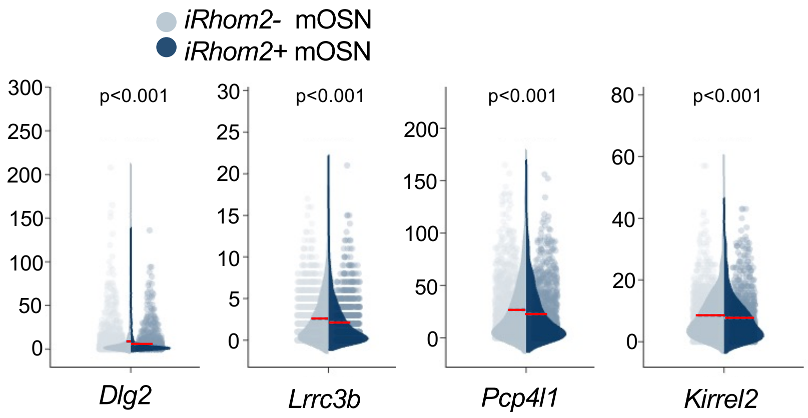

Supplementary Figure S6. Activity marker expression for *Dlg2*, *Lrrc3b*, *Pcp4l1*, and *Kirrel2* shows significantly higher expression in the iRhom2- OSNs versus the iRhom2+ OSNs. Results of Student’s t-tests are indicated.

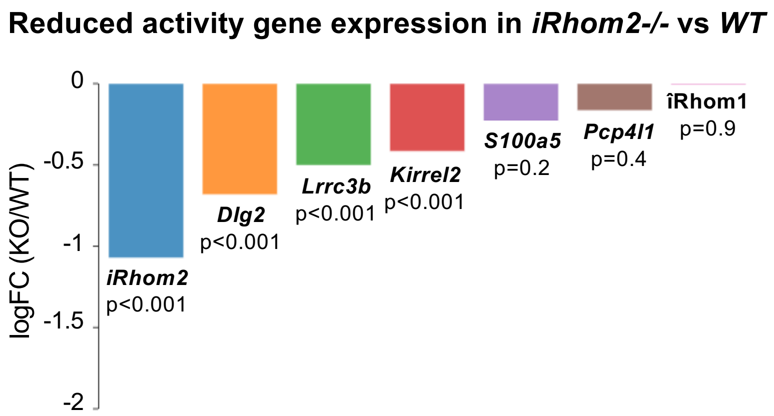

Supplementary Figure S7. Bulk RNAseq revealed reduced expression of OSN activity markers in *iRhom2-/-* OE (*n* = 12 per genotype). Results of Student's t-tests are indicated.

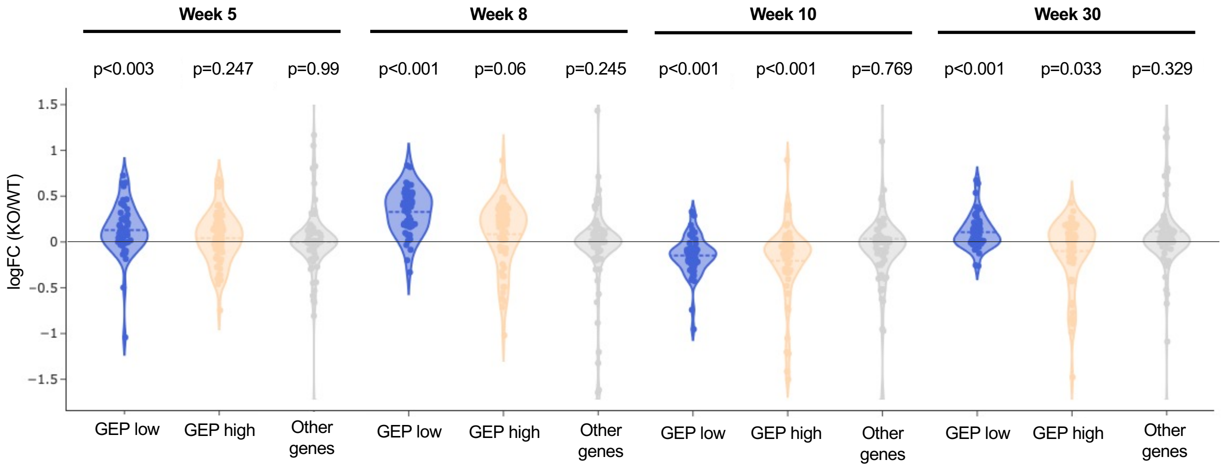

Supplementary Figure S8. GEP low genes [35] are significantly upregulated in bulk RNA Seq of *iRhom2-/-* OE at Week 5 (p=0.003), 8 (p<0.001), and 30 (p<0.001) compared to *WT*. GEP high genes [35] are downregulated in bulk RNAseq of *iRhom2-/-* OE at week 10 (p<0.001) and 30 (p=0.033). (*n* = 3 per group). Results of Student's t-tests are indicated.
